# A Comparison of the TempO-Seq S1500+ Platform to RNA-Seq and Microarray Using Rat Liver Mode of Action Samples

**DOI:** 10.3389/fgene.2018.00485

**Published:** 2018-10-30

**Authors:** Pierre R. Bushel, Richard S. Paules, Scott S. Auerbach

**Affiliations:** ^1^Biostatistics and Computational Biology Branch, NIEHS, Research Triangle Park, Durham, NC, United States; ^2^Biomolecular Screening Branch, National Toxicology Program, NIEHS, Research Triangle Park, Durham, NC, United States

**Keywords:** TempO-Seq, S1500+, microarray, RNA-Seq, mode of action, chemicals, toxicants, toxicogenomics

## Abstract

The TempO-Seq^TM^ platform allows for targeted transcriptomic analysis and is currently used by many groups to perform high-throughput gene expression analysis. Herein we performed a comparison of gene expression characteristics measured using 45 purified RNA samples from the livers of rats exposed to chemicals that fall into one of five modes of action (MOAs). These samples have been previously evaluated using Affymetrix^TM^ rat genome 230 2.0 microarrays and Illumina® whole transcriptome RNA-Seq. Comparison of these data with TempO-Seq analysis using the rat S1500+ beta gene set identified clear differences in the platforms related to signal to noise, root mean squared error, and/or sources of variability. Microarray and TempO-Seq captured the most variability in terms of MOA and chemical treatment whereas RNA-Seq had higher noise and larger differences between samples within a MOA. However, analysis of the data by hierarchical clustering, gene subnetwork connectivity and biological process representation of MOA-varying genes revealed that the samples clearly grouped by treatment as opposed to gene expression platform. Overall these findings demonstrate that the results from the TempO-Seq platform are consistent with findings on other more established approaches for measuring the genome-wide transcriptome.

## Introduction

High-throughput transcriptomics (HTT) is increasingly being adopted for screening in chemical and toxicological genomics in part due to advances in technological (i.e., direct from lysate transcriptomics) and greater efficiency (e.g., target screening using sentinel genes; Subramanian et al., 2017). The National Toxicology Program has pursued the development of the S1500+ gene set (Mav et al., 2018) screening platform utilizing the TempO-Seq^TM^ technology from BioSpyder^TM^ (Yeakley et al., 2017). Before there is widespread adoption of a new transcriptomic technology such as the TempO-Seq S1500+ platform, it will be important to establish its performance and degree of reproducibility compared to other more established techniques for gene expression assessment including microarray and whole transcriptome RNA-Seq. In addition to baseline performance issues such as signal to noise and identification of appropriate normalization procedures (Su et al., 2014), it is also critical to determine reproducibility of findings from established legacy platforms particularly in the case where large compendium data such as the Connectivity Map (Lamb et al., 2006) or the Toxicogenomics Project-Genomics Assisted Toxicity Evaluation System (Igarashi et al., 2015) have been generated and serve as means to interpret new findings derived from newer technologies such as TempO-Seq. In addition, it is important for biologists that stand-alone assessments of gene set enrichment yield valid findings consistent with established modes or mechanisms of action and scalability of machine learning classifiers established using older technology (Waters et al., 2010).

To address the absolute and relative performance metrics of the rat S1500+ beta gene set TempO-Seq platform we have measured the transcriptome of identical liver RNA samples from the DrugMatrix database that were used to evaluate the performance of whole transcriptome RNA-Seq compared to microarray toward the SEquence Quality Control (SEQC)/MicroArray Quality Control III (MAQC3) toxicogenomics study in which the transcripts from the latter two platforms were matched for a fair comparison (Gong et al., 2014). The training data set consists of 63 samples measured using TempO-Seq S1500+, Illumina® whole transcriptome RNA-Seq, and Affymetrix^TM^ Rat 230 2.0 microarrays. From the exposures of the rats to the chemicals, five different modes of action (MOAs) in the liver are represented in the samples including orphan nuclear hormone receptors (CAR/PXR) activation, aryl hydrocarbon receptor (AhR) activation, peroxisome proliferator-activated receptor alpha (PPARA) activation, cytotoxicity, and DNA Damage (Table 1). The treatments used vary considerably in their elicited transcriptomic signal (i.e., number of MOA-varying genes) and reveal degrees of distinctiveness in the altered gene sets which is ideal for establishing the level of granularity/resolution by which the technologies produce similarity in their resultant findings. Using the DrugMatrix samples we provide here a systematic comparison of the TempO-Seq technology relative to microarray and whole transcriptome RNA-Seq.

**Table 1:** Chemicals, modes of action, and exposures.

**MOA**	**Chemical**	**Dose (mg/kg body weight)**	**Duration (days)**	**Agent type**
Aryl hydrocarbon receptor (AhR)	3-Methylcholanthrene (3ME)	300	5	Carcinogen
	Leflunomide (LEF)	60	5	Antirheumatic drug
	beta-Naphthoflavone (NAP)	1,500	5	Putative chemopreventive agent
Orphan nuclear hormone receptors (CAR/PXR)	Phenobarbital (PHE)	54	5	Barbiturate drug
	Methimazole (MET)	100	3	Antithyroid drug
	Econazole (ECO)	334	5	Antifungal medication
Cytotoxicity (Cytotox)	Chloroform (CHO)	600	5	Organic compound
	Thioacetamide (THI)	200	5	Carcinogen
	Carbon tetrachloride (CAR)	1,175	7	Solvent for cleaning products, refrigerant
DNA Damage (DNA_Damage)	Aflatoxin B1 (AFL)	0.3	5	Mycotoxin
	Ifosfamide (IFO)	143	3	Chemotherapy drug
	N-Nitrosodimethylamine (NIT)	10	5	Organic compound
Peroxisome proliferator-activated receptor alpha (PPARA)	Pirinixic acid (PIR)	364	5	Hypolipidemic drug
	Bezafibrate (BEZ)	617	7	Hypolipidemic drug
	Nafenopin (NAF)	338	5	Hypolipidemic drug

## Materials and methods

### Samples and exposures

Mode of action (MOA) samples, preparation of them, RNA extraction and microarray and RNA-Seq analyses are as previously described (Wang et al., 2014). Briefly, male Sprague-Dawley rats (aged 6–8 weeks and weighing 200–260 g) were dosed once daily in triplicate for 3, 5, or 7 days, depending on the test chemical, and livers were harvested 24 h after the last dose. Animals were handled in accordance with the United States Department of Agriculture and Code of Federal Regulations Animal Welfare Act (9 CFR Parts 1, 2, and 3). Details on the design and in life portion of these studies can be found elsewhere. For each of the five MOAs there were three test chemicals (Table 1). RNAs from the treated rats were extracted and stored in the National Toxicology Program (NTP) DrugMatrix Frozen Tissue Library.

### Microarray analysis

cRNA was labeled and hybridized to the Affymetrix (Santa Clara, CA, United States) whole genome GeneChip® Rat Genome 230 2.0 Array as previously described (Wang et al., 2014). The arrays were scanned using the GeneChip Scanner 3000 7G and CEL files generated using the GeneChip Operating Software (GCOS). The data was then log_2_ transformed and normalized using the robust multichip average (RMA) algorithm (Irizarry et al., 2003a,b). The transformed/normalized data is available at the DrugMatrix ftp site (ftp://anonftp.niehs.nih.gov/drugmatrix/Affymetrix_data/Normalized_data_by_organ/). Raw data files and processed data in various file formats are available in the Gene Expression Omnibus (GEO) (Edgar et al., 2002; Barrett et al., 2013) under accession number GSE47875.

### RNA-Seq analysis

Poly-A RNA was extracted from each RNA sample, fragmented, adapter ligated and enriched by 15 polymerase chain reaction (PCR) cycles for library generation. The library size distribution was validated on the Agilent Bioanalyzer (Santa Clara, CA, United States) using a DNA 1000 kit. The final library was generated from a band between 200 and 500 bp with a peak at ~260 bp. Using Illumina TruSeq RNA Sample Preparation Kit and SBS Kit v3 (San Diego, CA, United States), samples were prepared for sequencing. Paired-end RNA-Seq cluster generation and sequencing by synthesis was performed using Illumina HiScan or HiSeq 2000 sequencers according to the manufacture's protocol. Depths of 30–130 million of paired 100 bp reads were generated for each sample. Details of the methods are as previously described (Wang et al., 2014). The raw data fastq files are available in the National Center for Biotechnology Information Sequence Read Archive (SRA; Leinonen et al., 2011) under accession number SRP039021.

### Preprocessing of RNA-Seq data

Alignment, quantification and normalization of the RNA-Seq data are as previously described (Wang et al., 2014). Briefly, RNA-Seq reads in fastq files were mapped using the Magic aligner (ftp://ftp.ncbi.nlm.nih.gov/repository/acedb/Software/Magic) to the following references:

- The *Rattus norvegicus* genome build RGSC v3.4
- The RefSeq and AceView 2008 (Thierry-Mieg and Thierry-Mieg, 2006) gene and transcript models, respectively
- Mitochondrial genes
- rRNA genes (manually constructed from multiple GenBank accessions, in the absence of RefSeq)
- External RNA Control Consortium (ERCC) RNA spike in control sequences (National Institute of Standards and Technology, Gaithersburg, MD, United States)
- A control genome constructed by complementing the *R. norvegicus* genome bases (i.e., exchange A:T and G:C), but not reversing the order. As such, the control genome has exactly the same composition as the reference genome but alignments to it are false positives and removed

No mismatch is reported closer than 8 bases to the edge of the aligned segment. Reads mapping to several alternative transcripts of the same gene are retained but counted only once. The read count for each transcript per sample was transformed and normalized as follows:

$$\text{Index}=log_{2}\left(Z+\;\sqrt{\left(4+Z^{2}\right)}\;\right)-1$$

Where *Z* $=10^{12}\left(\frac{n}{NL}\right)$, *n* is the read count of the transcript, *N* is the read depth for the sample and *L* is the length of the transcript. For transcripts that are not highly expressed (<3 read counts) the Index was imputed with 5.0. The preprocessed data (not imputed) is available in GEO under accession number GSE55347.

To match AceView transcripts from the RNA-Seq platform to probe sets on the Affymetrix microarray, each transcript sequence was mapped against the Affymetrix probes from each probe set using the Magic Aligner and allowing for a single-mismatch. Transcripts (*n* = 28, 975) mapping to at least 8 probes within a probe set unambiguously (meaning not mapping to any other probes from other probe sets) are considered a one-to-one match in terms of them being representative of the same transcript probe set. These were then mapped to UniGene (Pontius et al., 2002) cluster IDs (March 30, 2016) for Gene Ontology (GO) biological process (BP) enrichment analysis.

### TempO-Seq analysis

The sequencing library for the rat liver RNA samples (identical samples employed for RNA-Seq in the previously published SEQC toxicogenomics study Wang et al., 2014) was prepared by BioSpyder Technologies, Inc. (Carlsbad, CA, United States) according to their protocol guidelines. One microliter of each RNA sample (500–660 ng/uL) was hybridized with the S1500+ beta detector oligo pool mix (2 μl per sample) using the following thermocycler settings: 10 min at 70°C, followed by gradual decrease to 45°C over 49 min, and ending with 45°C for 1 min. Hybridization was followed by nuclease digestion (24 μl nuclease mix addition followed by 90 min at 37°C), ligation (24 μl ligation mix addition followed by 60 min at 37°C), then heat denaturation (at 80°C for 30 min). Ten microliters of each ligation product were then transferred to a 96-well PCR amplification microplate that also contained 10 μl of PCR mix per well. Through amplification well-specific, “barcoded” primer pairs were introduced to templates. Five microliters of the PCR amplification products from each well were then pooled into a single sequencing library. The TempO-Seq library was then processed with a PCR clean-up kit (Machery-Nagel, Mountain View, CA, United States) prior to sequencing. Sequencing was performed using a 50 cycle single-end read flow cell on a NextSeq 550 Sequencing System (Illumina, San Diego, CA, United States). Processing of sequencing data was conducted using Illumina's BCL2FASTQ software employing default parameter settings. Sequencing data were demultiplexed to generate fastq files and passed through internal quality controls. fastq files were analyzed using the TempO-SeqR software package (BioSpyder Technologies, Inc., Carlsbad, CA, United States). The raw data fastq files are available in the SRA under accession number SRP158667. The TempO-SeqR package maps reads from the fastq file using the Bowtie2-2.1.0 algorithm (Langmead et al., 2009) to a subset of the rat transcriptome (Refseq release 70 downloaded July 23rd 2015) reflecting the 50 nt sequences targeted by the detector oligos. Indels were not allowed, up to 2 base pair mismatches were allowed and multimapping of sequence reads was not allowed. The output of the TempO-SeqR package was a table of counts with each column representing a sample and each row representing a gene generated using the QuasR v1.8.4 Bioconductor package (Gaidatzis et al., 2015). The count data matrix is available in GEO under accession number GSE118956. Gene symbols were mapped to UniGene cluster IDs (June 6, 2015).

### Preprocessing of TempO-Seq data

Of the 2,284 genes targeted in the rat S1500+ beta gene set (NTP Tox21 S1500 Webpage: https://ntp.niehs.nih.gov/results/tox21/researchphases/index.html), those with a total read count ≤214 across all the samples were removed leaving 2,055 genes. The counts per gene were normalized to counts per million (CPM) by dividing it by the total read count per sample and multiply by 10^6^. The CPM normalized data was then transformed with log_2_ using an offset of 1.

### Log_2_ ratio values generation

For each gene/transcript in a data set, the average of the log_2_ normalized data for the control samples were subtracted from the log_2_ normalized data of each gene/transcript within a sample matched according to nutritional status of the vehicle (i.e., corn oil vs. other non-nutritive vehicles).

### Principal variance component analysis

Principal Variance Components Analysis (PVCA; Li et al., 2009) combines the use of principal component analysis (PCA) with variance components analysis (VCA) through mixed linear modeling of gene expression data with random effect terms that account for variation related to factors in the experimental design. The variance of each random effect is called a variance component. Briefly, given a general linear model where y = Xβ + e and y denotes gene expression observations, X is the design matrix, β is the known fixed effects parameter vector and e is the unexplained variation. However, if the experimental design contains random factor levels, the model becomes a mixed effect linear model y = Xβ + Zu + e, where in addition to the terms denoted in a fixed effect model, Z is the design matrix for random effects, u is the vector of unknown random-effect parameters, and e is the unobserved vector of independent and identically distributed (iid) Gaussian random errors.

Given that the variance of y is **V=ZGZ'** + **R**, **V** can be modeled by setting up the random effects design matrix **Z** and by specifying the variance-covariance structure for **G** and **R**. In usual variance component models, **G** is a diagonal matrix with variance components on the diagonal, each replicated along the diagonal corresponding to the design matrix **Z**. **R** is simply the residual variance component times the n x n identity matrix. Thus, the goal becomes finding a reasonable estimate of **G** and **R**. The method of restricted maximum likelihood (REML) is the standard procedure to accomplish this and was specified in the **lmer** function of the lme4 R package (R Development Core Team, 2012) for fitting linear mixed effects models (Bates et al., 2015).

The following steps comprise of PVCA:

- From a PxN (genes by samples) matrix of log_2_ ratio values, obtain the NxN correlation matrix
- Perform PCA on the correlation matrix to obtain eigenvalues
- Determine the first K principal components (PCs) to explain ≥58.76% of the variation in the data
- Fit all factors as random effects in a mixed linear model using the K PCs and REML to obtain unbiased estimates of variance
- Standardize the variance component estimates from the model
- Compute weighted proportions of the standardized variance component estimates. Here the weights are the proportions of variation explained by the PCs
- Compute weighted average proportions of the standardize variance component estimates by averaging model effects according to the proportion of total variance across all estimates including the residual

### Root mean squared distance

Root mean squared distance (RMSD) is a measure of the gene expression distance between pairs of biological replicates (Wang et al., 2014). The gene expression distance between biological replicates *x* and y is

$$RMSD_{xy}=\sqrt{\frac{\sum_{i=1}^{N}{\left(I_{ix}-I_{iy}\right)}^{2}}{N}}$$

where *I* is the log_2_ gene expression ratio of *i*th gene/transcript in the corresponding biological replicate, and *N* is the number of genes/transcripts on the gene expression platform. For each chemical, there are three biological replicates and for each MOA there are three chemicals. The pairwise RMSD measures (*n* = 36) between treated rats within a MOA were averaged. This MOA-RMSD can be interpreted as a measure of the difference among the chemicals within a MOA. To compare the three gene expression platforms, the five MOA-RMSD measures were averaged to give a Platform-RMSD.

### Mode of action ANOVA

To obtain genes from each platform that vary significantly by MOA, we modeled the gene expression data with a MOA analysis of variance (MOA-ANOVA)

$$\begin{array}{l} Y_{ijkl}=\mu +M_{i}+R_{j}+C{\left(M^{*}R\right)}_{ijk}+\varepsilon_{ijkl} \end{array}$$

where *Y*_*ijkl*_ represents the *l*th log_2_ ratio gene expression observation on the *i*th MOA (M), *j*th route (R) and *k*th chemical (C). μ is the grand mean for the whole experiment and ε_*ijkl*_ represents the random error. The errors are assumed to be normally and independently distributed with mean 0 and standard deviation δ for all measurements. Chemical is a random effect. Multiple testing correction was controlled at a false discovery rate (FDR) of 0.05 (Benjamini and Hochberg, 1995).

### Gene expression profile signal to noise

Let us denote each gene expression log_2_ ratio as *g*_*ij*_ where *i* indicates a MOA inter-group index from 1 to *m, j* is the MOA intra-group index from 1 to *n*_*i*_, *m* is the number of MOAs and *n*_*i*_ is the number of chemicals in *i*^th^ MOA inter-group. To evaluate a gene expression profile within a MOA, we calculate each MOA intra-group average $\bar{g_{i}}$ and sample variance ${s_{i}}^{2}$. We define a gene expression profile's signal as

$$S=\left\{\begin{array}{l} \;\max\left\{{\bar{g}}_{i}\right\},if\min\left\{{\bar{g}}_{i}\right\}\;>\;0\\ \;-\min\left\{{\bar{g}}_{i}\right\},elseif\max\left\{{\bar{g}}_{i}\right\}\;<\;0\\ \;\max\left\{{\bar{g}}_{i}\right\}-\min\left\{{\bar{g}}_{i}\right\}\;otherwise \end{array}\right\}$$

where 1 ≤ *i* ≤ *m*.

We then define a gene expression profile's noise as the square-root of the pooled variance

$$N=\sqrt{\frac{\sum_{i}^{m}\left[(n_{i}-1)\cdot s_{i}^{2}\right]}{\sum_{i}^{m}\left(n_{i}-1\right)}\sum_{i}^{m}\frac{1}{n_{i}}}$$

where the sample variance

$$s_{i}^{2}=\frac{\sum_{j}^{n_{i}}{\left(g_{ij}-{\bar{g}}_{i}\right)}^{2}}{n_{i}-1}.$$

From *S* and *N*, we define a gene expression profile's signal-to-noise ratio as *SNR* = *S/N*. We use Extracting Patterns and Identifying co-Expressed Genes (EPIG; Chou et al., 2007) to (1) obtain a gene expression profile's SNR statistics and (2) cluster gene expression profiles into significant (*p* < E10^−4^) co-expression patterns.

### Gene ontology subtrees to tag and annotate genes within a set

To compare each platform in terms of enrichment of the genes that vary by MOA, we used GO subtrees to tag and annotate genes (goSTAG) within a set (Bennett and Bushel, 2017). Briefly, for each list of genes that vary by MOA at FDR < 0.01, the gene symbols were mapped to the GO BPs of the genes they represent using version 3.4 of the GO database and the rat2302 database. The 1.01 version of the “goSTAG” Bioconductor package in R was used to perform enrichment of GO BP terms, clustering and subtree generation. The union of the enriched GO BP terms from all of the DEGs lists yielded 203 terms. BP terms which were not significant had missing *p*-values and were imputed with 1.0. –Log_10_ *p*-values, a min of 5 genes per GO BP, FDR < 0.05, Pearson correlation similarity metric and Ward algorithm for clustering, cluster slicing using correlation (*r*) of 0.1 and a minimum of 5 GO BP terms per cluster for subtree generation were used as input and parameters. Clusters (those with a 1– *r* ≥ 0.9) of GO BP terms (*n* ≥ 5) were labeled according to the node having the maximum number of paths to it within the GO BP subtree directed acyclic graph derived from the terms in the cluster.

## Results

### Study design and exposures

**Figure 1:**
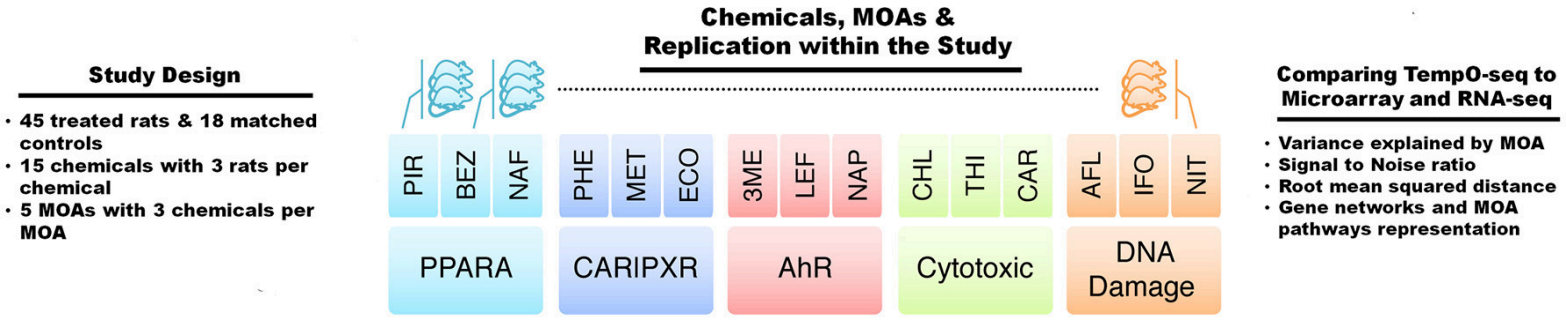
Study design. The study comprised of gene expression data acquired from male Sprague-Dawley rats dosed once daily in triplicate for 3, 5, or 7 days depending on the test chemical or matched control, and livers were harvested 24 h after the last dose. The abbreviations for the names of the chemicals are listed in Table 1. There were five modes of action (MOAs) with three chemicals per MOA. The MOAs are PPARA, peroxisome proliferator-activated receptor alpha; CAR/PXR, orphan nuclear hormone receptors; AhR, aryl hydrocarbon receptor; Cytotoxic, cytotoxicity, and DNA Damage. Comparisons between the data from TempO-Seq to microarray and RNA-Seq were performed by statistical and bioinformatics methodologies.

Gene expression analysis has advanced over the past 20+ years. Two main platforms for surveying genome-wide gene expression are microarray and RNA-Seq. Each of these platforms has its advantages and disadvantages (Lowe et al., 2017). The SEQC/MAQC3 consortium evaluated the concordance between Affymetrix microarray and Illumina RNA-Seq using toxicogenomics gene expression data (Wang et al., 2014). We used the SEQC/MAQC3 study design to compare the two aforementioned platforms (with transcripts matched between the two) with the TempO-Seq platform targeting the rat S1500+ beta gene set. As shown in Figure 1, the study design consists of rats exposed in triplicate to 45 chemical or controls whereby three of the chemicals share one of five MOAs: PPARA, CAR/PXR, AhR, cytotoxicity, and DNA damage. The chemicals, the MOA that each one represents, exposure doses and durations and the types of agents are listed in Table 1. The doses and durations of the exposures were selected to ensure a maximal transcriptional response. Animals were dosed once daily for 3, 5, or 7 days, depending on the chemical. Livers were harvested 24 h after the last dose, RNA samples extracted and then prepared for gene expression analysis. We used three statistical strategies and bioinformatics tools to examine GO BPs, metabolic pathways and BP subnetworks for comparison of the three platforms.

### Specifications of the platforms

Table 2 details some general specifications of the three gene expression platforms. The Affymetrix rat whole genome microarray with >31,000 gene probe sets uses *in situ* hybridization for interrogation of gene expression. The *de facto* normalization procedure is RMA. The Illumina RNA-Seq next generation HiScan or HiSeq 2000 platforms were used. They measure gene expression by nucleotide chain termination sequencing by synthesis. Although at this time there is no standard approach for bioinformatics analysis of RNA-Seq data, we used the AceView transcriptome gene model and Magic normalization index that performed the best among several bioinformatics pipelines in the SEQC/MAQC3 consortium evaluation (Wang et al., 2014). In addition, 28, 975 transcripts from the two aforementioned platforms were matched bioinformatically (see the Materials and Methods section) to assure a one-to-one mapping. Finally, BioSpyder's rat S1500+ beta TempO-Seq platform differs from RNA-Seq in that it uses templated oligonucleotides representative of >2,200 Refseq genes to sequence captured RNA templates. Filtering by total read counts retained 2,055 genes (see the Materials and Methods section). We used CPM for normalization. The data from all three platforms were log_2_ transformed to make the data more normally distributed.

**Table 2:** Platforms used for comparison.

**Gene expression type**	**Microarray**	**RNA-Seq**	**TempO-Seq**
Platform	Affymetrix whole genome GeneChip Rat Genome 230 2.0	Illumina HiScan & HiSeq 2000	BioSpyder S1500+ Beta
Technology	*In situ* oligonucleotide array	Next generation nucleotide chain termination sequencing by synthesis	Templated oligonucleotide detection
Gene content/gene model	~31,000 gene probe sets	~38,100 AceView transcripts	~2,200 Refseq genes
Normalization	RMA	Magic normalized index	TPM
Transformation	Log_2_	Log_2_	Log_2_

### Variance components of the study design captured by the platforms

**Figure 2:**
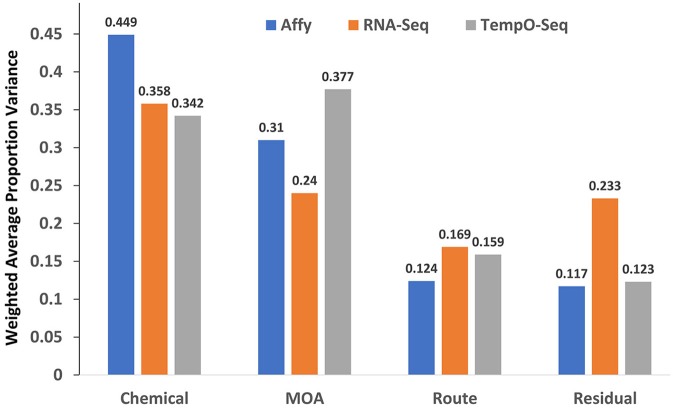
Variance components explained. Shown on the y-axis is the weighted average of the proportion of variance explained by platform for each of the mixed effect linear model terms denoted in the x-axis.

The study design contained factors that represents the chemical used for exposure, the MOA of the chemical and the route of the exposure. We performed PVCA on the normalized and log_2_ transformed data from each platform to determine which captured the most variation in gene expression. As shown in Figure 2, the microarray platform captured slightly more variance related to the chemical used for treatment (0.449), but the TempO-Seq platform captured variation related to the MOA (0.377) slightly more than the other two platforms. It seems that the RNA-Seq platform had more unexplained variation captured as residuals. This was not related to the two different Illumina sequencers used (data not shown). Route showed no difference in the variation captured by the three platforms.

### Expression pattern magnitude of change and signal to noise revealed by each platform

**Figure 3:**
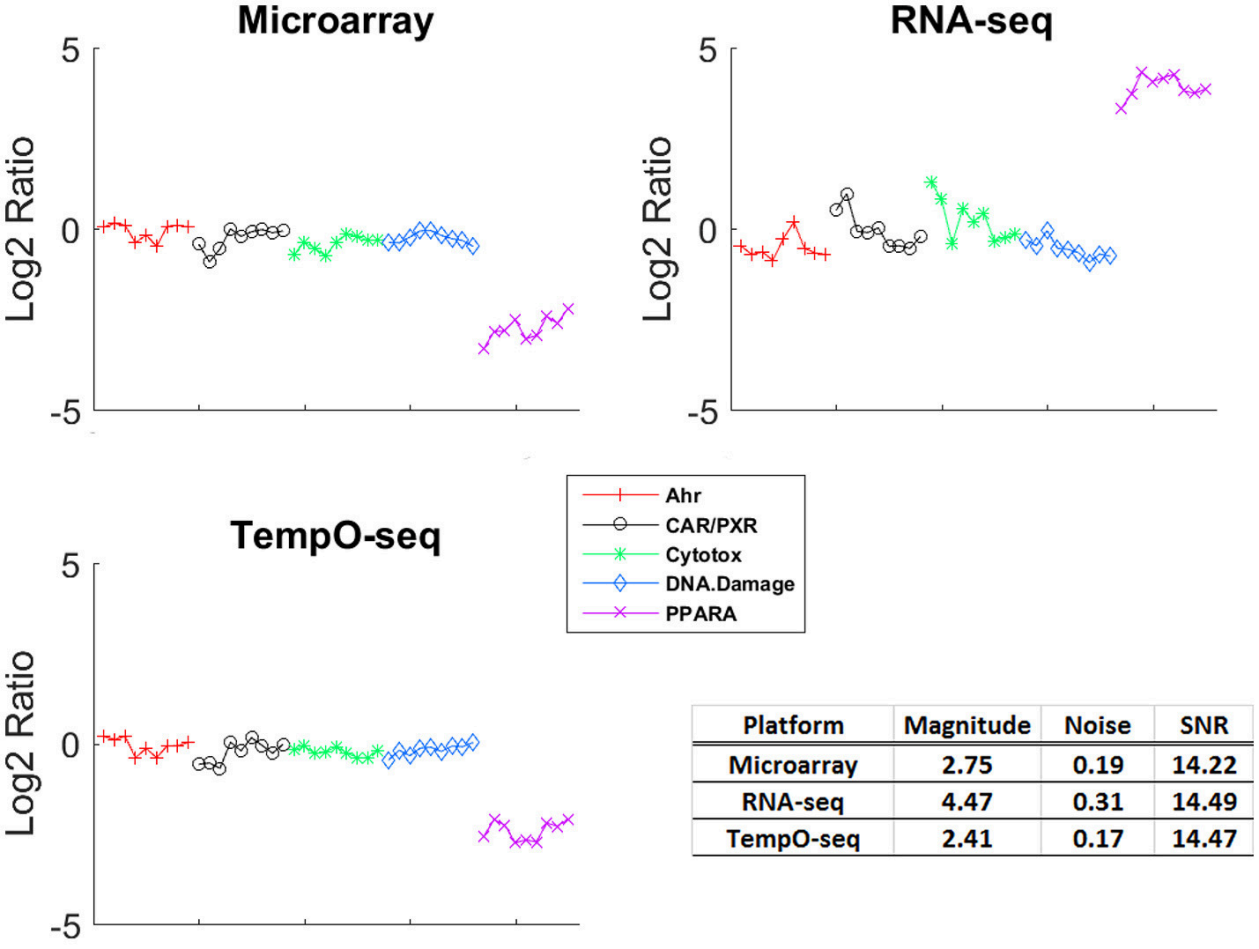
Gene expression patterns with maximal signal to noise. For each platform, the EPIG pattern with the maximal signal to noise ratio (SNR) is shown. The y-axis is the log_2_ ratio of gene expression (treated to the average of the control [matched according to nutritional status of the vehicle]), the x-axis is the samples grouped by MOA (represented by the colors and symbols in the legend). The table inset displays the magnitude of fold change, the noise and the SNR for each of the patterns shown.

One of the more informative ways to compare gene expression data is to assess the magnitude of change and the SNR of a response. To compare the gene expression from the three platforms, we analyzed the data using EPIG which used magnitude of fold change, correlation and SNR to categorize gene expression profiles into co-expressed patterns (Chou et al., 2007). Shown in Figure 3 is the pattern of gene expression from each platform that had the maximal magnitude of fold change relative to control. The samples were grouped by MOA. Although RNA-Seq had the highest magnitude of fold change (4.47), the noise of the expression profiles that made up the pattern is higher (0.31) than the other two platforms. When all the patterns for each platform were taken into consideration, the average SNR was substantially higher for TempO-Seq than the other two platforms (Table 3). This may be related to the EPIG analysis of the TempO-Seq data yielding only four patterns whereas microarray yielded 17 and RNA-Seq yielded 11 (data not shown).

**Table 3:** Replication agreement and signal to noise within platform.

**Measure**	**Microarray**	**RNA-Seq**	**TempO-Seq**
Ave. Chemical-RMSD	0.33	0.98	0.71
Platform-RMSD	0.42	1.11	0.93
Average SNR	6.6	6.9	9.14

### Cohesiveness of replicate gene expression by platform

A unique design of the study is that there is replication at the animal level, the chemical level, and the MOA level (Figure 1). We harnessed this feature to assess how well each platform captured similar gene expression between replicates. We used RMSD to assess the gene expression distance between pairs of biological replicates. A smaller measure means the replicates are closer to each other in terms of gene expression. The platform-RMSD is an aggregate (overall average) of the distance between animals treated with a chemical, the chemicals within a MOA and the five MOAs. The average chemical-RMSD is the mean of the RMSDs for each chemical by platform. As shown in Table 3, the Platform-RMSD and average chemical-RMSD were more than 2 times lower for microarray than for the other two platforms. RNA-Seq had the highest RMSD (1.11 at the platform level and 0.98 at the chemical level) which may be related to the higher noise level seen in the expression pattern for this platform (Figure 3). Despite the relatively noisy RNA-Seq platform, PCA of the gene expression data revealed that RNA-Seq captured a higher percent of the variability (55.5%) in the data than the other two platforms and also projected the samples in 3-dimensional space closer to each other in terms of MOA (Data not shown).

### Biological responsiveness by platform

**Figure 4:**
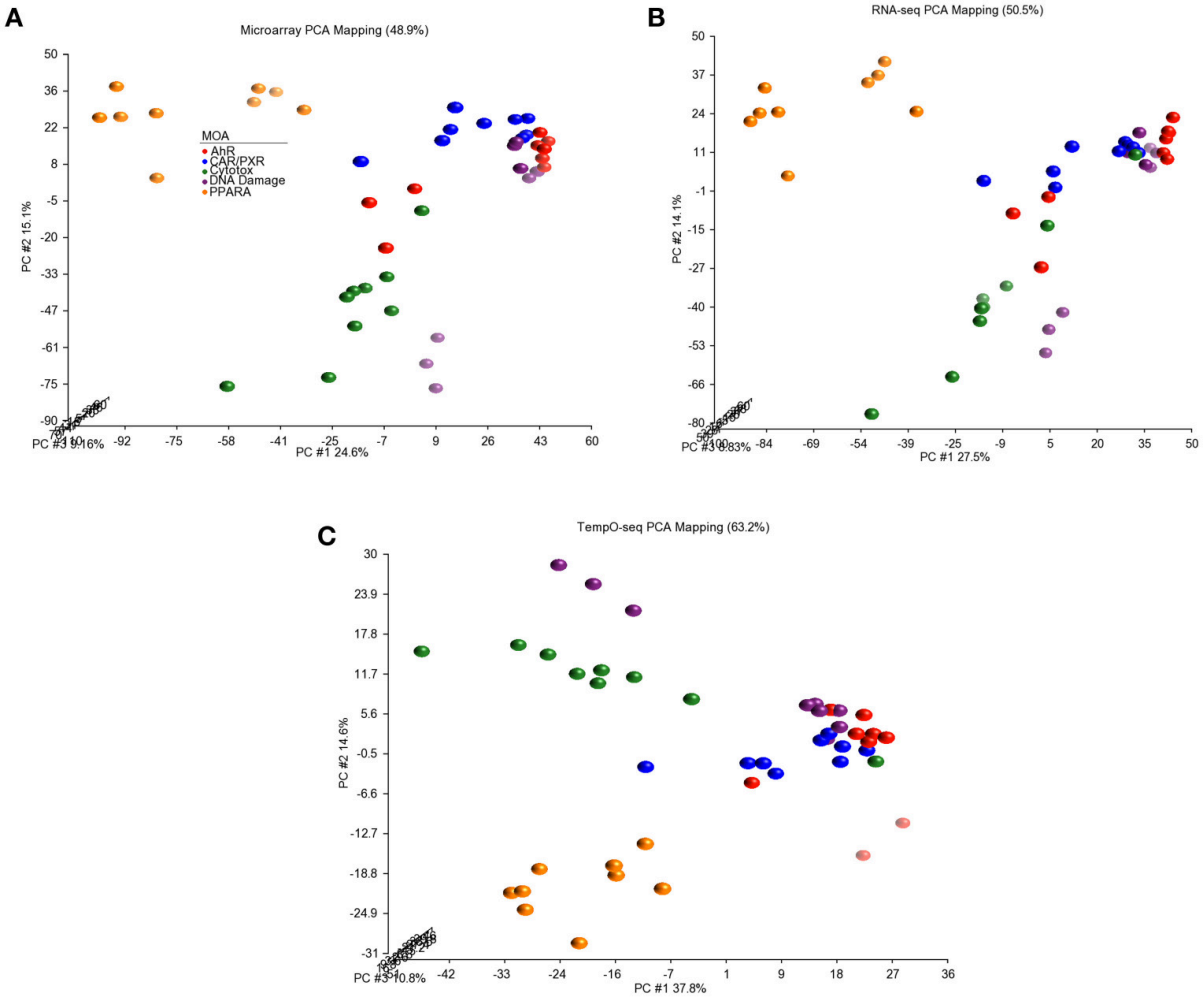
Principal component analysis of the data. **(A)** Microarray. **(B)** RNA-Seq. **(C)** TempO-Seq. PCA performed using the log_2_ ratio expression data (treated to matched control according to nutritional status) of the genes that vary by MOA at FDR < 0.01.

**Figure 5:**
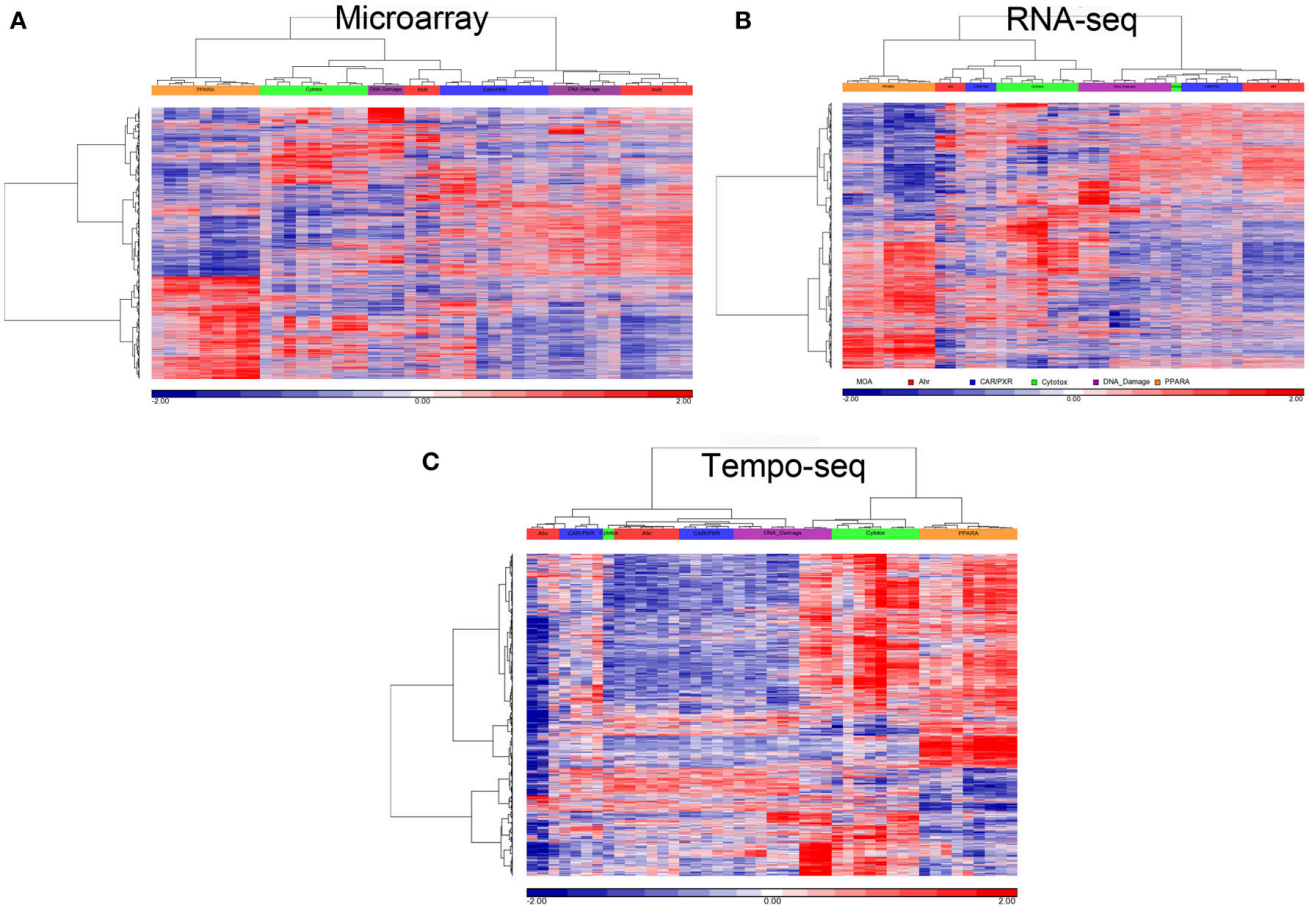
Clustering of data. **(A)** Microarray. **(B)** RNA-Seq. **(C)** TempO-Seq. Clustering performed using the log_2_ ratio expression data (treated to matched control according to nutritional status) of the genes that vary by MOA at FDR < 0.01 with cosine correlation as the similarity metric and the Ward clustering criterion. The data for clustering was standardized to a mean of 0 and standard deviation of 1. Samples' MOA colored as in the legend to Figure 4A.

Since three chemicals share a MOA, for each platform we used an ANOVA model with MOA as a main factor to identify genes that vary significantly at an FDR < 0.01. For microarray, RNA-Seq and TempO-Seq, 9,499 probe sets, 7,217 transcripts and 1,366 genes were detected as varying, respectively (Supplemental Table 1). These genes should drive the clustering of the gene expression data by MOA. As shown in Figures 4, 5, respectively, PCA and 2-dimensional hierarchical clustering of the data from each platform by their MOA varying genes was reasonably good. However, each platform had at least two MOAs with a chemical that didn't cluster with its respective MOA chemicals. For microarray NIT and LEF didn't cluster with DNA damage and AhR MOA chemicals, respectively. For RNA-Seq LEF, ECO and one biological replicate of CAR didn't cluster with AhR, CAR/PXR, and cytotoxicity MOA chemicals, respectively. For TempO-Seq LEF and ECO didn't cluster with Ahr, and CAR/PXR MOA chemicals, respectively. In addition, one biological replicate of MET and CAR and didn't cluster with their biological replicates in the CAR/PXR and cytotoxicity MOA chemicals, respectively.

**Figure 6:**
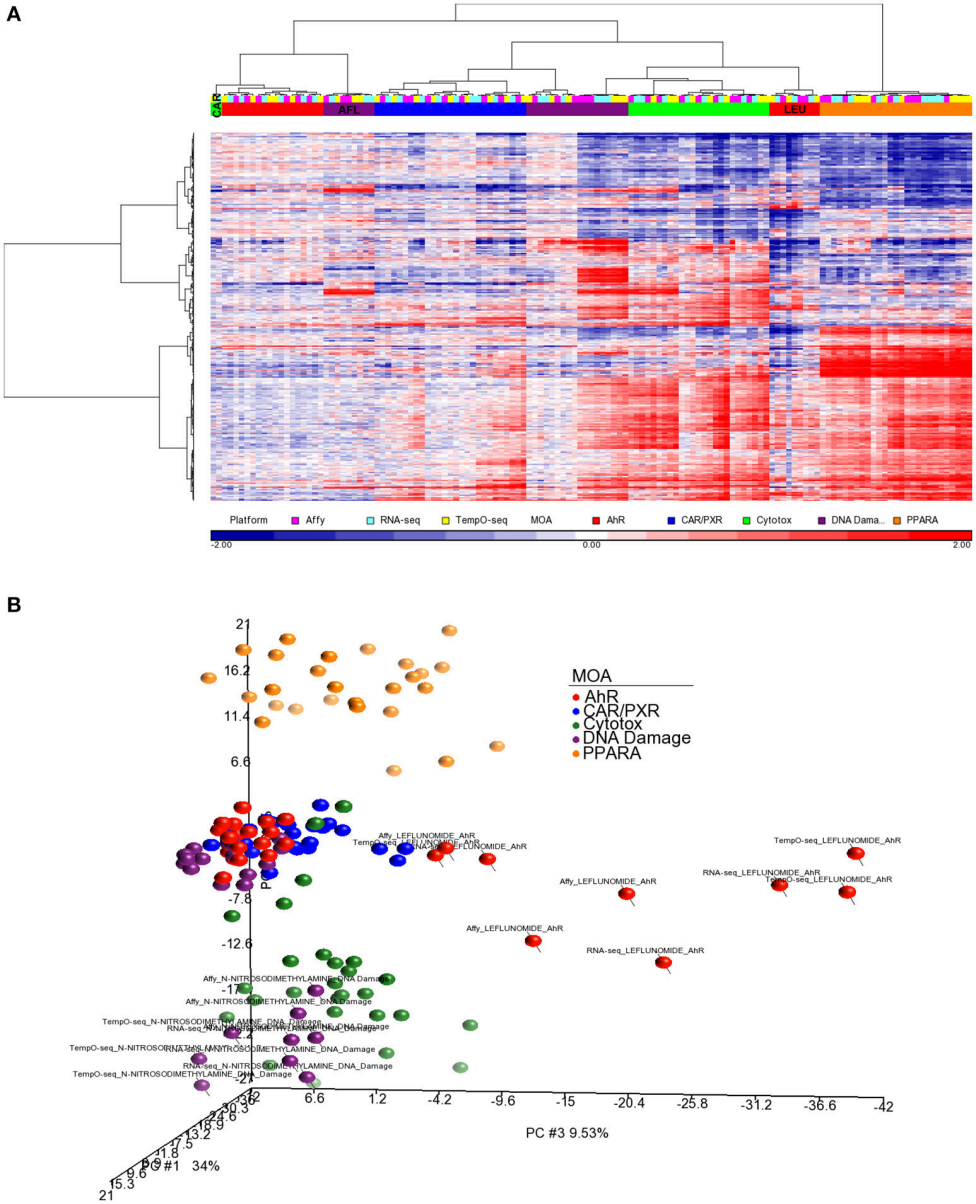
Clustering of the data using a common gene set. **(A)** Hierarchical clustering performed using the log_2_ ratio expression data (treated to matched control according to nutritional status) of the genes that vary by MOA at FDR < 0.01 and map to 731 UniGene cluster IDs that overlap between the three platforms. Genes that were mapped to the same UniGene cluster ID were averaged. The cosine correlation was used as the similarity metric and the Ward clustering criterion for merging clusters. Samples' MOA colored as in the legend to Figure 4A. Platforms are represented by the following colors: pink, Affymetrix; light blue, RNA-Seq; yellow, TempO-Seq. **(B)** PCA of the data used in **(A)**. Principal component (PC) #1 = 34%, PC #2 = 16.6 %, and PC #3 = 9.53.

For better cluster resolution, we mapped the MOA varying genes from each platform to UniGene cluster IDs and then compiled the log_2_ ratio data from all three platforms using the 731 UniGene cluster IDs that overlapped (Supplemental Table 2). Genes that were mapped to the same UniGene cluster ID were averaged. As shown in Figure 6A, the clustering of the samples was mostly by MOA except for LEF, AFL and one biological replicate from CAR. PCA of the data captures ~60% of the variation in the data and projected the samples in 3-dimensional space closer to each other in terms of MOA except for LEF and NIT samples from all three platforms (Figure 6B).

**Figure 7:**
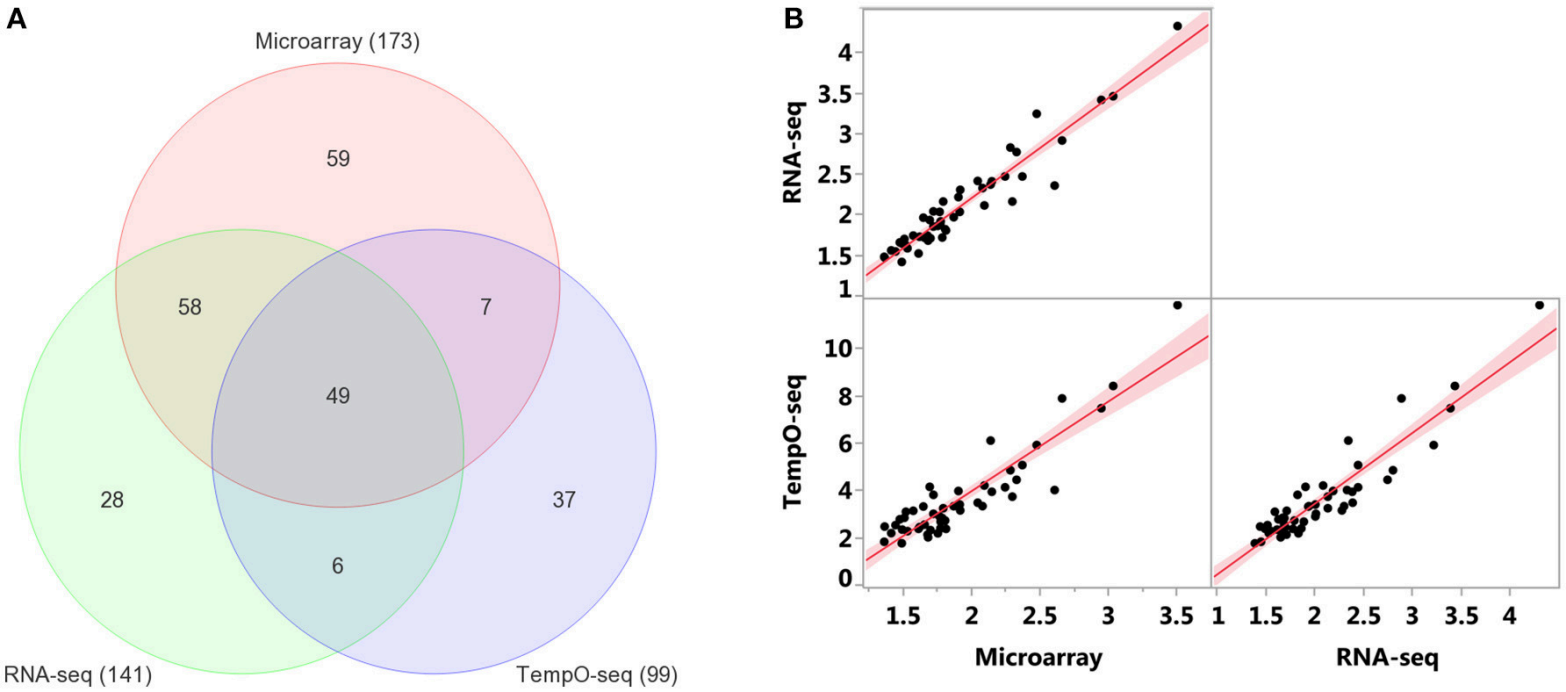
Comparison of enriched GO biological processes (BPs). **(A)** Overlap of enriched GO BPs < FDR 5%. Minimum number of genes = 3 for TempO-Seq and 5 for the other two. **(B)** Pairwise comparison of GO BPs fold enrichment from the 49 categories in common between the three platforms. Red line is the linear fit (regression line) with 95% level confidence boundaries.

Enrichment of GO biological processes by the platforms' varying genes yielded 49 significant categories (FDR < 5%) that overlapped (Figure 7A). Microarray had the most enriched categories (*n* = 173), followed by RNA-Seq (*n* = 141), and then TempO-Seq (*n* = 99). Some of the enriched GO biological processes that overlapped related to fatty acid metabolism, apoptosis, liver development, and lipid metabolism (Table 4). As shown in Figure 7B, the correlation of the 49 GO biological processes fold enrichment between the three platforms was very high (*r* > +0.9).

**Table 4:** Enriched GO BPs (FDR < 5%) that overlap between platforms^®^.

**GOID**	**GO BP Term**	**Microarray**				**RNA-seq**				**TempO-seq**			
		**Count**	**%**	**Pop Hits**	**FE**	**Count**	**%**	**Pop Hits**	**FE**	**Count**	**%**	**Pop Hits**	**FE**
GO:0001666	Response to hypoxia	119	2.11	270	1.55	99	2.18	270	1.59	39	3.32	270	2.28
GO:0001731	Formation of translation preinitiation complex	17	0.30	24	2.50	18	0.40	24	3.24	9	0.77	24	5.92
GO:0001889	Liver development	85	1.51	145	2.07	81	1.78	145	2.42	32	2.72	145	3.48
GO:0006413	Translational initiation	32	0.57	52	2.17	29	0.64	52	2.41	13	1.11	52	3.95
GO:0006446	Regulation of translational initiation	19	0.34	31	2.16	17	0.37	31	2.37	12	1.02	31	6.11
GO:0006457	Protein folding	57	1.01	112	1.79	49	1.08	112	1.89	17	1.45	112	2.40
GO:0006629	Lipid metabolic process	44	0.78	89	1.74	42	0.93	89	2.04	17	1.45	89	3.01
GO:0006631	Fatty acid metabolic process	33	0.58	60	1.94	32	0.71	60	2.31	12	1.02	60	3.16
GO:0006635	Fatty acid beta-oxidation	35	0.62	46	2.68	31	0.68	46	2.92	23	1.96	46	7.89
GO:0006637	Acyl-coa metabolic process	19	0.34	28	2.39	16	0.35	28	2.47	9	0.77	28	5.07
GO:0006695	Cholesterol biosynthetic process	17	0.30	26	2.30	17	0.37	26	2.83	8	0.68	26	4.86
GO:0006749	Glutathione metabolic process	31	0.55	52	2.10	28	0.62	52	2.33	11	0.94	52	3.34
GO:0006915	Apoptotic process	157	2.78	366	1.51	120	2.64	366	1.42	41	3.49	366	1.77
GO:0006953	Acute-phase response	25	0.44	38	2.32	19	0.42	38	2.16	9	0.77	38	3.74
GO:0006979	Response to oxidative stress	76	1.35	146	1.83	61	1.34	146	1.81	22	1.87	146	2.38
GO:0007568	Aging	150	2.66	315	1.68	125	2.75	315	1.72	51	4.34	315	2.56
GO:0007584	Response to nutrient	69	1.22	137	1.77	59	1.30	137	1.86	19	1.62	137	2.19
GO:0007623	Circadian rhythm	52	0.92	121	1.51	46	1.01	121	1.64	18	1.53	121	2.35
GO:0009636	Response to toxic substance	65	1.15	119	1.92	61	1.34	119	2.22	30	2.55	119	3.98
GO:0009749	Response to glucose	54	0.96	106	1.80	47	1.04	106	1.92	18	1.53	106	2.68
GO:0010033	Response to organic substance	72	1.28	152	1.67	69	1.52	152	1.96	32	2.72	152	3.32
GO:0010243	Response to organonitrogen compound	35	0.62	68	1.81	34	0.75	68	2.16	14	1.19	68	3.25
GO:0014070	Response to organic cyclic compound	138	2.45	272	1.79	128	2.82	272	2.04	50	4.26	272	2.90
GO:0031100	Organ regeneration	45	0.80	91	1.74	39	0.86	91	1.85	22	1.87	91	3.82
GO:0031667	Response to nutrient levels	49	0.87	112	1.54	42	0.93	112	1.62	22	1.87	112	3.10
GO:0032355	Response to estradiol	91	1.61	201	1.60	81	1.78	201	1.74	40	3.40	201	3.14
GO:0032496	Response to lipopolysaccharide	114	2.02	280	1.43	101	2.23	280	1.56	39	3.32	280	2.20
GO:0032869	Cellular response to insulin stimulus	66	1.17	123	1.89	56	1.23	123	1.97	26	2.21	123	3.34
GO:0033539	Fatty acid beta-oxidation using acyl-coa dehydrogenase	16	0.28	19	2.97	15	0.33	19	3.42	9	0.77	19	7.48
GO:0042493	Response to drug	246	4.36	528	1.64	211	4.65	528	1.73	82	6.98	528	2.45
GO:0042542	Response to hydrogen peroxide	40	0.71	78	1.81	31	0.68	78	1.72	14	1.19	78	2.83
GO:0043065	Positive regulation of apoptotic process	133	2.36	338	1.39	115	2.53	338	1.47	53	4.51	338	2.47
GO:0043066	Negative regulation of apoptotic process	203	3.60	517	1.38	177	3.90	517	1.48	60	5.11	517	1.83
GO:0043434	Response to peptide hormone	63	1.12	129	1.72	51	1.12	129	1.71	19	1.62	129	2.32
GO:0045471	Response to ethanol	82	1.45	193	1.50	74	1.63	193	1.66	34	2.89	193	2.78
GO:0046686	Response to cadmium ion	27	0.48	45	2.11	22	0.48	45	2.12	12	1.02	45	4.21
GO:0051289	Protein homotetramerization	37	0.66	76	1.72	34	0.75	76	1.94	20	1.70	76	4.15
GO:0051301	Cell division	83	1.47	179	1.63	63	1.39	179	1.52	27	2.30	179	2.38
GO:0051384	Response to glucocorticoid	69	1.22	133	1.83	56	1.23	133	1.82	23	1.96	133	2.73
GO:0051603	Proteolysis involved in cellular protein catabolic process	28	0.50	51	1.93	24	0.53	51	2.04	11	0.94	51	3.40
GO:0055088	Lipid homeostasis	27	0.48	42	2.27	24	0.53	42	2.47	11	0.94	42	4.13
GO:0055114	Oxidation-reduction process	314	5.56	651	1.70	262	5.77	651	1.74	88	7.49	651	2.13
GO:0070542	Response to fatty acid	26	0.46	39	2.35	25	0.55	39	2.77	11	0.94	39	4.45
GO:0071407	Cellular response to organic cyclic compound	53	0.94	122	1.53	48	1.06	122	1.70	22	1.87	122	2.85
GO:0071456	Cellular response to hypoxia	57	1.01	137	1.47	49	1.08	137	1.55	22	1.87	137	2.53
GO:0097421	Liver regeneration	41	0.73	55	2.63	30	0.66	55	2.36	14	1.19	55	4.02
GO:0098609	Cell-cell adhesion	102	1.81	211	1.70	82	1.81	211	1.68	27	2.30	211	2.02
GO:1904871	Positive regulation of protein localization to Cajal body	8	0.14	8	3.52	8	0.18	8	4.33	6	0.51	8	11.84
GO:1904874	Positive regulation of telomerase RNA localization to Cajal body	13	0.23	15	3.05	12	0.26	15	3.46	8	0.68	15	8.42

® *Universe is 17,535. List totals: Microarray: 4,976; RNA-Seq: 4,053; TempO-Seq: 1,111. Enrichment performed using UniGene cluster IDs and the Database for Annotation, Visualization and Integrated Discovery (DAVID) v6.8*.

**Figure 8:**
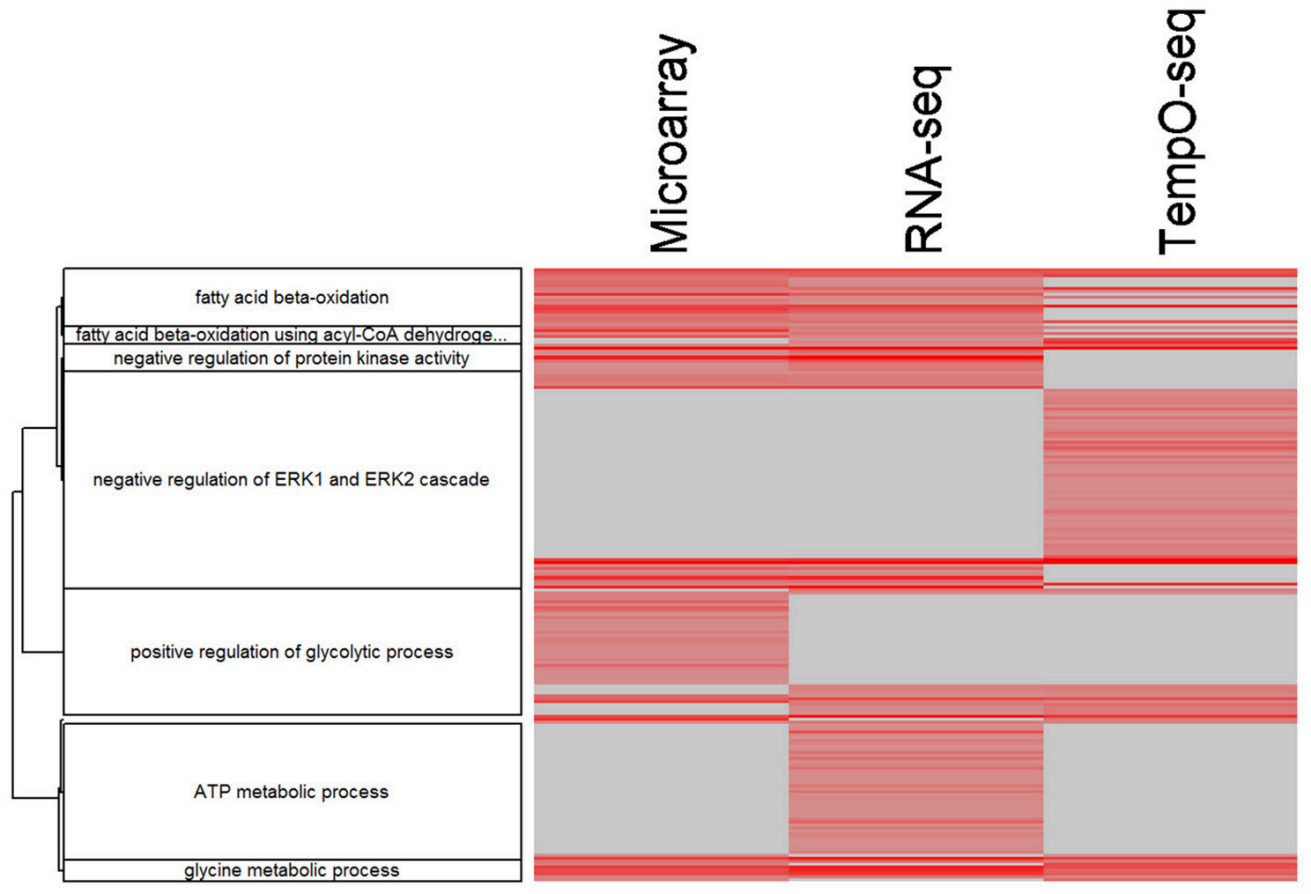
Clustering of enriched GO BPs. goSTAG clustering of 203 enriched GO BPs using 5 genes per category, BH FDR < 0.05, correlation distance (1-Pearson correlation) and Ward clustering, dendrogram threshold = 0.9 and minimum number of GO BP terms per cluster = 5. Data is the –log_10_ *p*-value. The more red the intensity, the more significant the enrichment. Gray indicates that the GO BP term was not enriched significant and thus the *p*-value was imputed with 1.0.

Comparing and contrasting gene set enrichments can be challenging when there are many categories to consider. To more formally compare the three platforms in terms of biology, we used goSTAG to identify subtrees of enriched GO BPs from the MOA varying genes and then find the categories that are shared or differ between platforms. As shown in Figure 8, all three platforms enriched for subtrees that map to fatty acid beta-oxidation and glycine metabolic process. However, TempO-Seq enriched for subtrees that map to negative regulation of ERK1 and ERK2 cascade. RNA-Seq uniquely enriched subtrees that map to ATP metabolic process and microarray exclusively enriched for subtrees that map to positive regulation of glycolytic process.

## Discussion

Over the last two decades gene expression analysis has advanced to permit genome-wide transcriptomics. Affymetrix microarray and Illumina RNA-Seq are two platforms that have gained popularity for gene expression analysis. Each has its own advantages and disadvantages but currently, the platform of choice for gene expression analysis seems to be RNA-Seq.

Comparison of the two platforms was performed using liver RNA samples from rats exposed to chemicals that have particular modes of action (MOAs; Wang et al., 2014; Figure 1 and Table 1). We used the microarray and RNA-Seq training data from these samples to compare with the data generated from the samples using the TempO-Seq platform. TempO-Seq is unique in that the platform's gene content (~2,200) consists of bioinformatically curated (Mav et al., 2018) and expert domain-nominated rat genes that represent the totality of biological perturbation space (Table 2). This makes the TempO-Seq platform very appealing for transcriptomics in that (1) sequencing of the RNA is from sample lysates negating the need for library construction which are large sources of variability in RNA-Seq (Su et al., 2014), (2) the sequencing cost is much less than RNA-Seq given the number of targeted templates and pooling of samples in a multiplexed sequencing run allowing for more resources to expand experimental designs, and (3) the data storage is reasonable and the bioinformatics more simplified leading to a quicker turn-around in results and data analysis manageable by a wider group of analysts.

An obvious disadvantage to the TempO-Seq platform is that the gene content is predefined requiring extensive template fabrication and careful probe sequence curation. Furthermore, the targeted sequencing design renders TempO-Seq incapable of discerning novel transcripts. Despite these shortcomings, TempO-Seq performed similarly to microarray and RNA-Seq with respect to analysis of the SEQC/MAQC3 MOA toxicogenomics data. When statistical parameters were used to evaluate the three platforms, TempO-Seq had comparable variance structure related to chemical treatment, MOA and route of administration (Figure 2). Not surprisingly, we observed that RNA-Seq had higher unexplained variance (Figure 2), a larger noise component in expression patterns (Figure 3), and greater error between biological replicates (Table 3). This might be due to the large variation in lowly expressed genes that RNA-Seq detects at high sequencing depths. When the top percentile of expressed genes from RNA-Seq were used to evaluate expression differences between biological replicates, the error was much lower than when additional lower expressed genes were used (Wang et al., 2014). TempO-Seq variation, noise, and error in gene expression was moderate, falling between microarray and RNA-Seq.

Since each transcript profiling platform has different numbers of gene content and annotation, we explored the ability of each to cluster the samples by using the set of genes that vary statistically by MOA. We used an ANOVA model for each data set with chemical, MOA and route as the main effects. For microarray, RNA-Seq and TempO-Seq, 9,499 probe sets, 7,217 transcripts, and 1,366 genes were detected as significantly (FDR < 0.01) varying, respectively (Supplemental Table 1). These MOA-varying genes and those mapped to 731 UniGene cluster IDs (Supplemental Table 2) as a common set were used for cluster analysis. In both cases the clustering of the samples by MOA for each platform was similar in that at most two chemicals from two MOAs were not clustered with their respective MOA chemicals (Figures 5, 6A). In addition, the clustering of the samples by PCA with platform-specific MOA-varying genes mapped to the common UniGene set projected the samples into 3-dimensional space (Figure 6B) representative of the MOAs similar to the outcome when just MOA-varying genes from each platform were used (Figure 4). Hence, it is plausible that the TempO-Seq platform with the reduced gene content set is sufficient to resolve gene expression space elicited by a wide variety of chemical stressors with distinct MOAs. Utilization of the TempO-Seq platform for evaluation of chemicals using gene expression suggests that the platform may gain popularity in biomolecular screening efforts in the near future (Grimm et al., 2016; House et al., 2017).

It has been proven that reproducibility between gene expression is higher when the data are compared on the pathway level than the gene level (Guo et al., 2006; Fan et al., 2010; Wang et al., 2014). We enriched the MOA-varying UniGenes according to GO BPs and revealed that the reduced representation of genes on the TempO-Seq platform had a negligible effect on the overrepresentation (Figure 7 and Table 4). This is in line with the bioinformatics process to select the S1500+ sentinel gene content on the platform using diversity and co-expression importance scores (Mav et al., 2018). These genes were selected to cover >90% of the biological pathway space represented by MSigDB (Subramanian et al., 2005). Yet each platform does appear to have enrichment of unique BPs as depicted in GO subtrees of overrepresented biological categories (Figure 8).

Having another tool for biologists to survey genome-wide gene expression is a luxury for scientific experimentation. With microarray fully matured and easy to analyze, and RNA-Seq flexible to interrogate complex transcriptional machinery, scientists have diverse platforms to investigate biological consequences that regulate gene expression genome-wide. The emerging TempO-Seq platform adds to the genomics tool chest and with comparable performance capabilities to its predecessors, will undoubtedly play a pivotal role in high-throughput screening efforts.

## Author contributions

PB conceptualized the analysis strategy, performed the analyses, interpreted the results, and wrote parts of the paper. SA provided the samples that the data were generated from, interpreted the results, provided biological, toxicological context, and wrote parts of the paper. RP helped to interpret the results and provided biological, toxicological context.

### Conflict of interest statement

The authors declare that the research was conducted in the absence of any commercial or financial relationships that could be construed as a potential conflict of interest.
